# PROVIDER AND SYSTEM IMPACTS OF THE UW ECHO IN RURAL AND FRONTIER CARE TRANSITIONS

**DOI:** 10.1093/geroni/igz038.1083

**Published:** 2019-11-08

**Authors:** Catherine Carrico, Tonja Woods, Robin A Barry, Kevin Franke, Christine McKibbin

**Affiliations:** 1 University of Wyoming, Laramie, Wyoming, United States; 2 Mountain Pacific Quality Health, Casper, Wyoming, United States

## Abstract

Background: The Project ECHO model utilizes a hub and spoke approach through which a team of experts co-mentors local providers in the management of complex cases while disseminating information about best practices and evidence-based care. Project ECHO is a promising model for improving patient care through transformation of the care delivery system. The UW ECHO in Rural and Frontier Care Transitions created an online community of practice comprised of local care coalitions dedicated to improving care transitions in Wyoming and Montana. This ECHO network provided a unique opportunity to support system- and provider-level implementation of best practices in care transitions. Methods: Semi-structured interviews were conducted with thirty ECHO attendees following participation in an ECHO session as either a participant or case presenter. Thematic analysis was used to analyze interview data. Results: Two overarching themes emerged 1) impact of the ECHO on the provider or healthcare team and 2) impact on the system. Participants indicated that the impact on the provider/healthcare team included an increased sense of community, increased awareness of community resources, increased knowledge of care transition strategies, and increased confidence in implementing best practices. Additionally, providers indicated increased utilization of community resources. Systemic impacts included increased involvement of interprofessional team members in patient care and utilization of ECHO recommendations to present systemic interventions and changes to colleagues, administration, and leadership. Conclusions: This ECHO network had a particularly strong impact on the provider and healthcare team as participants increased their knowledge, confidence, and use of best practices in care transitions.

